# Stage-specific requirement for METTL3-dependent m^6^A modification during dental pulp stem cell differentiation

**DOI:** 10.1186/s12967-022-03814-9

**Published:** 2022-12-16

**Authors:** Haiyun Luo, Wenjing Liu, Yachuan Zhou, Yanli Zhang, Junrong Wu, Ruolan Wang, Longquan Shao

**Affiliations:** 1 Stomatological Hospital, Southern Medical University, 366 Jiangnan Avenue South, Guangzhou, 510280 China; 2 State Key Laboratory of Oral Diseases & National Clinical Research Center for Oral Diseases & Department of Cariology and Endodontics, West China Hospital of Stomatology, Sichuan University, Chengdu, 610041 China

**Keywords:** RNA methylation, Cell differentiation, Stem cell therapy, Poly(A) tail length

## Abstract

**Background:**

N^6^-methyladenosine (m^6^A) is the most prevalent epigenetic modification in eukaryotic messenger RNAs and plays a critical role in cell fate transition. However, it remains to be elucidated how m^6^A marks functionally impact the transcriptional cascades that orchestrate stem cell differentiation. The present study focuses on the biological function and mechanism of m^6^A methylation in dental pulp stem cell (DPSC) differentiation.

**Methods:**

m^6^A RNA immunoprecipitation sequencing was utilized to assess the m^6^A-mRNA landscape during DPSC differentiation. Ectopic transplantation of DPSCs in immunodeficient mice was conducted to verify the in vitro findings. RNA sequencing and m^6^A RNA immunoprecipitation sequencing were combined to identify the candidate targets. RNA immunoprecipitation and RNA/protein stability of Noggin (NOG) were evaluated. The alteration in poly(A) tail was measured by 3′-RACE and poly(A) tail length assays.

**Results:**

We characterized a dynamic m^6^A-mRNA landscape during DPSC mineralization with increasing enrichment in the 3′ untranslated region (UTR). Methyltransferase-like 3 (METTL3) was identified as the key m^6^A player, and METTL3 knockdown disrupted functional DPSC differentiation. Moreover, METTL3 overexpression enhanced DPSC mineralization. Increasing m^6^A deposition in the 3′ UTR restricted NOG expression, which is required for DPSC mineralization. This stage-specific m^6^A methylation and destabilization of NOG was suppressed by METTL3 knockdown only in differentiated DPSCs. Furthermore, METTL3 promotes the degradation of m^6^A-tagged NOG by shortening the poly(A) tail length in the differentiated stage.

**Conclusions:**

Our results address an essential role of dynamic m^6^A signaling in the temporal control of DPSC differentiation and provide new insight into epitranscriptomic mechanisms in stem cell-based therapy.

**Supplementary Information:**

The online version contains supplementary material available at 10.1186/s12967-022-03814-9.

## Introduction

Mesenchymal stem cells (MSCs) residing in various tissues can still undergo self-renewal and differentiate into specific cell types to maintain tissue homeostasis and fulfill regenerative needs [1]. Multiple adult stem cell types with diverse biological properties have been reported in many tissues and organs. Beyond their similarity in cellular and molecular functions, MSCs exhibit distinct features related to their original phenotypes. Dental pulp stem cells (DPSCs) have superior pluripotency capacity and high mineralization potential, which are essential for hard tissue formation [2]. DPSCs can differentiate into odontoblasts and secrete mineralized matrix known as tertiary dentin bridge formation to seal the vital pulp chamber and prevent pulpal infection from potential insult [3]. The differentiation of DPSCs is critical for tertiary dentin formation and dental repair in vital pulp therapy [3, 4]. Meanwhile, accumulating evidence also supports that DPSCs are capable of differentiating into osteoblasts and forming lamellar bone, which is a promising stem cell source in bone engineering [5]. The initiate signaling reprograms cellular differentiation and extracellular matrix secretion will greatly benefit therapeutic approaches in engineering DPSC-based vital pulp procedures. Epigenetic modulations are capable of temporally controlling the transcription programs in a heritable manner and subsequently guiding quiescent stem cells to undergo preferential trajectories toward differentiation [6]. The key epigenetic regulators could reversibly modulate endogenous stem cell activities and promote functional mineralized tissue formation, which present therapeutic opportunities in regenerative strategies.

N^6^-methyladenosine (m^6^A) is the most prevalent posttranscriptional modification of messenger RNA (mRNA) and regulates almost every step of RNA metabolism in mammals [7]. The methyltransferase complex formed by methyltransferase-like 3 (METTL3), METTL14, and Wilms tumor 1-associated protein (WTAP) is responsible for the m^6^A modification of mRNA, which catalyzes adenosines with methyl groups from metabolite substrates [8]. Large-scale transcripts are dynamically and timely tagged by m^6^A marks to orchestrate the different stages of stem cells. RNA m^6^A modification has emerged as a critical epitranscriptomic mechanism that regulates embryonic development, cell reprogramming and differentiation [9, 10]. The disruption of m^6^A modification in the stem cell program displays diverse effects across cell types and specific fate stages. Reduction of m^6^A deposition improved pluripotency and blocked regeneration of embryonic stem cells [11], while it limited self-renewal and triggered cell differentiation in epiblast stem cells and induced pluripotent stem cells [12, 13]. It is critical to identify the regulatory mechanism of dynamic m^6^A marks in the DPSC fate transition. Our previous study characterized the m^6^A-tagged landscape in immature DPSCs, which is related to cell senescence and apoptosis [14]. Meanwhile, how m^6^A methylation participates in DPSC differentiation remains unclear. Clarifying the RNA epigenetic mechanism during DPSC differentiation and manipulating the key modulators in therapeutic applications would advance vital pulp therapy.

In this study, we revealed a dynamic and unique m^6^A-mRNA landscape with m^6^A RNA immunoprecipitation-sequencing (m^6^A RIP-seq), which provides an entry point to uncover the potential function of m^6^A methylation in DPSC differentiation. METTL3 was identified as a key molecule that mediates m^6^A modification in DPSC mineralization. Remarkably, dynamic m^6^A methylation of noggin (NOG) confers its stabilization by shortening the poly(A) tail in a stage-specific manner. Our results provide evidence for the critical role of m^6^A modification in DPSC differentiation and shed light on the epitranscriptomic mechanism in the temporal control of cell fate transition.

## Materials and methods

### DPSC isolation and culture

Dental pulp tissues were collected according to the appropriate guidelines after written informed consent was obtained by a protocol approved by the Human Research Committee of Stomatological Hospital, Southern Medical University (ethical code 2019(16)). Primary DPSCs were harvested and cultured as previously described [15]. Briefly, dental pulp tissues were removed from extracted third molars and then digested with 3 mg/mL collagenase I (Gibco-Invitrogen, Carlsbad, CA, USA). DPSCs were collected and cultured in Dulbecco’s modified Eagle’s medium (DMEM) supplemented with 10% fetal bovine serum and 1% penicillin and streptomycin (all from Gibco-Invitrogen) at 37 °C with 5% CO_2_. The medium was changed every 2 days, and DPSCs at passages 3–5 were used for subsequent experiments.

DPSCs (at a density of 2 × 10^5^ cells/mL) were seeded in 6-well or 24-well plates (Corning Life Sciences, NY, US) and cultured until the cells reached 80–90% confluence. The culture medium was changed to osteo/odontogenic medium (OM) containing 10 mmol/L β-glycerophosphate, 50 μg/mL ascorbic acid and 0.1 μmol/L dexamethasone (all from Sigma‒Aldrich, St. Louis, MO, USA). The induction medium was changed every other day from day 0 to day 21 (the day of induction was defined as day 0). After 7 days of induction, the cells were fixed and stained for alkaline phosphatase (ALP) (Beyotime Biotechnology, Shanghai, China) (n = 5). Alizarin red staining (ARS) (Beyotime) was used to stain the accumulated mineralized matrix on day 14 (n = 5). For adipogenic differentiation, DPSCs were induced by the Adipogenesis Differentiation Kit (HUXXC-90031, Cyagen Biosciences, Guangzhou, China). Cells were cultured with adipogenic inducing solution A for 3 days, and the medium was replaced with solution B for 1 day and then replaced with solution A. This cycle was repeated four times and then subjected to oil red O staining (Cyagen Biosciences) (n = 5).

### Total m^6^A and methylated metabolite measurements

DPSCs were subjected to osteo/odontogenic induction for 0, 7, and 14 days, and RNA was extracted using TRIzol solution (Takara Biotechnology, Shiga, Japan). Total m^6^A was measured with an m^6^A RNA methylation quantification kit (P-9019-96, Epigentek, Farmingdale, NY, US) according to the manufacturer’s protocol.

Liquid chromatography with tandem mass spectrometry (LC‒MS/MS) analysis was utilized to assess the metabolite compounds related to m^6^A methylation, including S-adenosylmethionine (SAM) and S-adenosylhomocysteine (SAH). Cell samples were subjected to methanol and homogenized before analysis with an ultrahigh-performance liquid chromatography (UHPLC) column (1290 Infinity LC, Agilent Technologies). Next, during MS/MS acquisition, a m/z range of 25–1000 Da was used, and the ion accumulation time was screened. Metabolite compounds were identified with a database of available standards and subjected to multivariate data analysis. Metabolites with a variable importance in projection (VIP) value > 1 were further subjected to statistical analysis.

### m^6^A RIP-seq and m^6^A RIP-qPCR

m^6^A RIP-seq was used to characterize m^6^A modification during DPSC mineralization (osteo/odontogenic medium-induced DPSCs, OM-DPSCs). Total RNA was isolated from DPSCs induced for 0, 7, and 14 days with TRIzol solution. The extracted RNA enriched and purified with oligo(dT)-attached magnetic beads and an m^6^A RIP kit (17-10499, Millipore, Burlington, MA, US) according to the kit’s protocol. The purified m^6^A-RIP RNA fragments were then fragmented into small pieces with fragmentation buffer for sequencing. The RNA fragments were incubated with magnetic beads conjugated with an m^6^A-specific antibody in buffer. The IP RNA and input RNA were reverse transcribed into cDNA and subjected to deep sequencing on an Illumina NovaSeq™ 6000 platform for m^6^A RIP-seq [16]. m^6^A peak calling, distribution, motif mapping and enrichment analysis were performed by LC-BIO Technologies Co., Ltd. (Hangzhou, China). To identify specific genes targeted by METTL3, the enrichment of m^6^A-modified *Noggin (NOG)* mRNA in the immunoprecipitate (IP) and input RNA was quantified by qPCR analysis as described previously for m^6^A RIP-qPCR [14].

### RNA sequencing and profile analysis

Total RNA was extracted from DPSCs, and poly(A) mRNA was purified with poly(T)-conjugated magnetic beads. Then, the mRNA was fragmented into small pieces and converted into double-stranded cDNA. Paired‐end runs with a read length of approximately 300 base pairs (bp) were used for RNA‐sequencing with the Illumina deep sequence platform by LC-BIO Technologies Co., Ltd. Significantly differentially expressed genes (DEGs) were identified in this study as genes with a fold change in expression ≥ 2.0 and corresponding *p* value < 0.05. The DEGs were subjected to Gene Ontology (GO) and Kyoto Encyclopedia of Genes and Genomes (KEGG) pathway enrichment analyses with the R packages GOseq and DAVID.

### Lentiviral vector construction and infection

Two independent METTL3 shRNA sequences (shMETTL3-1 and shMETTL3-2) within lentiviral vectors were used. shMETTL3-1 lentivirus encoding METTL3-shRNA and shCTR-1 lentivirus encoding scrambled control (shCTR) sequences were constructed by GeneChem Company (Shanghai, China); shMETTL3-2 and shCTR-2 were purchased from Santa Cruz Biotechnology (Santa Cruz, CA, US). cDNA encoding the full-length METTL3 gene was amplified and used to construct a lentiviral vector for METTL3 overexpression (LV-METTL3) by GeneChem Company. For lentivirus transfection, DPSCs were transfected with lentivirus at a multiplicity of infection (MOI) of 50 and cultured for 72 h before subsequent experiments. DPSCs with METTL3 knockdown were treated with 2 µg/mL human Noggin peptide (ab16380, Abcam, Cambridge, UK) to neutralize noggin protein during DPSC differentiation.

### Quantitative polymerase chain reaction (qPCR)

The cells were digested and total RNA was extracted from DPSCs with a Total RNA Isolation Kit (Foregene Biotechnology, Chengdu, China). The RNA was then reverse transcribed with RT Master Mix (Takara) according to the manufacturer’s instructions to obtain complementary DNA (cDNA). Real-time qPCR was performed with TB Green qPCR Mix (Takara) according to the manufacturer’s protocol. Relative target gene expression was analyzed with a standard curve and normalized to Glyceraldehyde-3-Phosphate Dehydrogenase (*GAPDH*) expression. The primer sequences used in qPCR are summarized in Additional file 1: Table S1.

### Western blot analysis

DPSCs were lysed and assayed with a BCA protein assay kit (Beyotime). Samples containing 15–30 μg of protein were subjected to sodium dodecyl sulfate polyacrylamide gel electrophoresis, and the proteins were then transferred to polyvinylidene fluoride membranes (Millipore). The membranes containing the transferred proteins were blocked with 5% bovine serum albumin and reacted with the primary antibody overnight at 4℃. The membranes were then labeled with corresponding secondary antibody of horseradish peroxidase (HRP)-conjugated anti-mouse IgG or anti-rabbit IgG for 1 h at room temperature before visualization with SuperSignal enhanced chemiluminescence substrate (Thermo Fisher Scientific, Waltham, MA, US). Primary and secondary antibodies against the following proteins were used in this study: METTL3 (96391, 1:1000); METTL14 (51104, 1:1000); WTAP (56501, 1:1000); p-Smad1/5 (9516, 1:1000) were purchased from Cell Signaling Technology (CST, Danvers, MA, USA). NOG (sc-293439, 1:1000); RUNX Family Transcription Factor 2 (RUNX2, sc-390351, 1:1000); Dentin Sialophosphoprotein (DSPP, sc-73632, 1:1000); Smad1/2/3 (sc-7960, 1:1000); p-Smad3 (sc-517575, 1:1000) were obtained from Santa Cruz Biotechnology. GAPDH (60004-1-Ig, 1:3000); goat anti-mouse IgG (SA00001-1, 1:3000) and goat anti-rabbit (SA00001-2, 1:3000) were purchased from ProteinTech Group (ProteinTech, Wuhan, China).

### Animal model construction

The animal experiments were conducted in compliance with ARRIVE guidelines and the National Institutes of Health Guide for the Care and Use of Laboratory Animals. All procedures followed protocols approved by the Ethics Committees of Stomatological Hospital, Southern Medical University (ethical code 2019(16)). For ectopic transplantation studies, porous beta-tricalcium phosphate/hydroxyapatite (β-TCP/HA) discs (diameter: 4 mm, thickness: 2 mm) were obtained from Biological Materials Manufacturing Core, Sichuan University. Approximately 1 × 10^6^ transfected DPSCs were seeded on β-TCP/HA discs and cultured in a 24-well plate with odontogenic medium for 24 h [17, 18]. The composites of DPSCs and the β-TCP/HA scaffold were transplanted into the subcutaneous dorsal pockets of 6-week-old BALB/c immunodeficient nude mice (n = 10) [18, 19]. Two subcutaneous pockets were made on the right and left side of the dorsal surface, each allowing for one composite. The shCTR and shMETTL3 groups were carefully transplanted into the left and right subcutaneous regions, respectively (n = 5), as were the LV-METTL3 and LV-CTR groups (n = 5). After 4 weeks, the harvested implants were fixed with paraformaldehyde, followed by decalcification with Ethylenediaminetetraacetic acid (EDTA) for 2 weeks. The formation of new mineralized tissue was evaluated by Masson-trichrome staining.

### Immunofluorescence staining

The composites of DPSCs and the β-TCP/HA scaffold were fixed, dehydrated, embedded in paraffin and then cut at 5 µm. The tissue slides were subjected to immunofluorescence with a standard protocol. The slides were incubated with primary antibody overnight at 4℃. After washing, samples were interacted with the corresponding secondary HRP-conjugated antibody (1:1000, ProteinTech) for 1 h and then labeled with Cy3 Tyramine (11065, AAT Bioquest, Inc. Sunnyvale CA, US) or AF 488 Tyramide reagent (11070 AAT Bioquest). Primary antibodies against the following proteins were used in this study: METTL3 (15073-1-AP, 1:200, ProteinTech); NOG (sc-293439, 1:200, Santa Cruz); p-Smad3 (sc-517575, 1:100, Santa Cruz) and Smad1/2/3 (sc-7960, 1:100, Santa Cruz).

Immunofluorescence staining of the induced DPSCs was performed as described in a previous study [15]. Fixed cells were blocked and incubated with the primary antibodies anti-METTL3 and anti-NOG overnight and then the corresponding fluorescent secondary antibody of Cy3–conjugated anti-rabbit IgG (SA00009-2, 1:500, ProteinTech) or fluorescein (FITC)–conjugated anti-mouse (SA00003-1, 1:500, ProteinTech). Fluorescent images showing subcellular expression were obtained with a confocal microscope (LSM 900; Zeiss, Oberkohen, Germany).

### m^6^A site-specific mutant plasmid construction

To investigate the biological effect of the m^6^A methylated sites in the 3′ untranslated region (UTR) of NOG, full-length NOG cDNA was constructed and cloned into the NheI- and BamHI-digested Flag-PCDNA3.1(+) vector by Genecreate Biotechnology Co., Ltd. (Wuhan, China). The vector used to express NOG with the wild-type m^6^A motif was referred to as NOG-WT. Two different mutants (NOG-MUT1 and NOG-MUT2) were generated by introducing single-nucleotide mutations (A to T) in the four m^6^A motifs of the 3′ UTR after mapping the conserved m^6^A motif sequence in m^6^A RIP-seq. To examine mRNA expression, 293T cells were transfected with NOG-WT vector or a mutant NOG vector and subjected to qPCR [20].

### RNA and protein stability analysis

RNA transcription in DPSCs was inhibited by treatment with 5 µg/mL actinomycin D (ActD, 7240-37-1, Sigma‒Aldrich) as described in a previous study to analyze mRNA decay rates [21]. mRNA was isolated after 0, 4, and 8 h and subjected to qPCR. The half-life of NOG mRNA has been reported.

Protein translation in DPSCs was inhibited by treatment with 100 µg/mL cycloheximide (CHX, A8244, APExBIO Technology, Houston, TX, US) to analyze protein stability. Total protein was isolated after 0, 4, and 8 h and subjected to western blot analysis. The protein expression level of NOG was used to analyze its stability.

### 3′-Rapid amplification of cDNA ends (3′-RACE)

3′-RACE to obtain the 3′ UTR sequences of NOG from shMETTL3 or shCTR DPSCs after osteogenic induction was performed with a 3′-RACE kit (6106, Takara) according to the protocol. The 3′-RACE products after two rounds of amplification were purified and subcloned into a vector, and the amplified fragments were further identified by sequencing [22]. The *NOG*-specific primers used for 3′-RACE analysis in this study were as follows: 5′-CATGGTGTGCAAGCCGTCCAAGTC-3′, 5′-TCACGGTGCTGCGGTGGCGCTGTC-3′.

### Poly(A) tail assay

A poly(A) tail assay was performed with a Poly(A) Tail-Length Assay Kit (764551KT, Thermo Fisher) according to the protocol. Briefly, poly(A) polymerase was used to add G and I to the 3′ ends of the RNA, and the newly tailed RNA was converted to cDNA by reverse transcription. Then, *NOG*-specific forward and reverse primers and universal reverse primers were used to generate a product consisting of *NOG* with a poly(A) tail. The PCR products from METTL3 knockdown DPSCs in differentiated/undifferentiated stages were separated on agarose gels [23]. The specific primers for *NOG* were F: 5′-TAACCTGCTATTTATATTCCAGTGCCCTTC-3′ and R: 5′-TGAACTCTATAGCTTCTTCGAGGTCCAA-3′.

### Quantification and statistical analysis

The experiments in this study were carried out biologically repeated at least three times, and the data are presented as the mean ± standard deviation. Statistical differences were evaluated by one‑way analysis of variance (ANOVA) and corresponding post hoc tests for multiple comparisons. Unpaired two-tailed Student’s t test was used to compare two groups. A *p* value < 0.05 was considered to be statistically significant and was analyzed by GraphPad Prism 7.0 (La Jolla, CA, US).

## Results

### The dynamic m^6^A epitranscriptomic landscape in DPSC differentiation

**Fig. 1:**
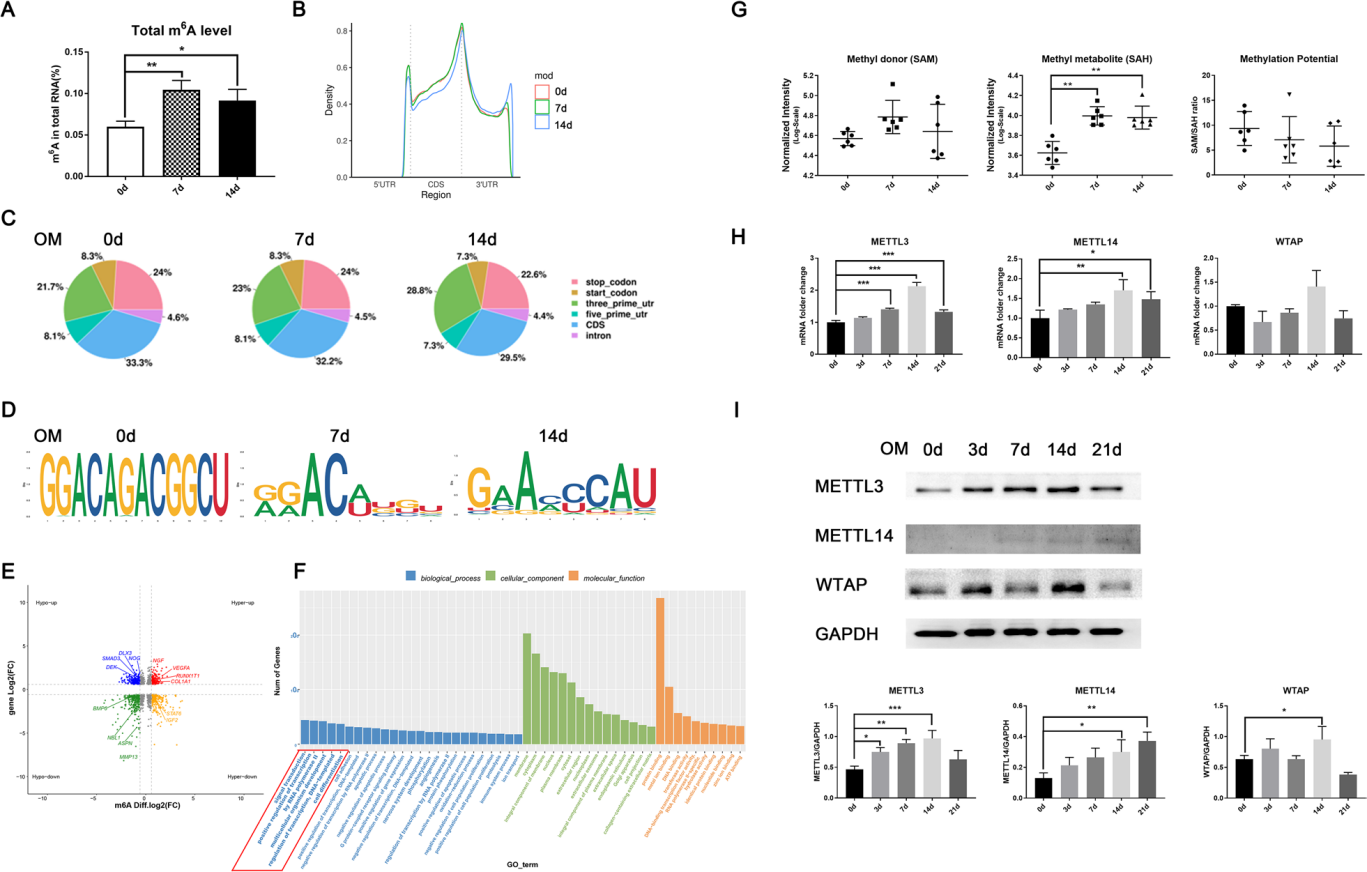
The dynamic m^6^A epitranscriptomic landscape in DPSC differentiation. **A** DPSCs were induced with osteo/odontogenic medium for 0, 7, and 14 days, and then the total m^6^A level in the RNA polls was quantified by the colorimetric method. **B** The m^6^A methylation landscape in the genome-wide transcriptome was evaluated by m^6^A RIP-seq. Distribution of m^6^A peaks in the 3′ UTR, CDS region and 5′ UTR. **C** Pie chart analysis of the m^6^A peak fraction in transcript segments. **D** Specific and conserved motif sequences of high-confidence m^6^A peaks identified by the HOMER database. **E** Epitranscriptome profile of m^6^A-tagged mRNAs in DPSCs after odontogenic differentiation. **F** Gene Ontology enrichment analysis of differentially m^6^A methylated and expressed transcripts. **G** The concentrations of SAM, SAH and potential of m^6^A establishment were qualified by LC‒MS/MS. Metabolites with a VIP value > 1 were further subjected to statistical analysis (n = 6). **H** The mRNA expression of METTL3, METTL14, and WTAP during DPSC mineralization, as evaluated by qPCR (n = 3). **I** The protein expression of METTL3, METTL14, and WTAP in DPSCs was evaluated by western blotting. SAM: S-adenosylmethionine; SAH: S-adenosylhomocysteine. Significance was determined via ANOVA or Student’s t test. The quantitative data are presented as the mean ± SD *p < 0.05. **p < 0.01. ***p < 0.001

We first examined the total m^6^A content and epitranscriptomic profile of m^6^A-tagged mRNA. The total m^6^A level in the RNA pool was upregulated upon osteo/odontogenic induction for 7 and 14 days, as quantified by a colorimetric method (Fig. 1A). m^6^A RIP-seq analysis showed that the majority of m^6^A peaks were enriched in the DNA coding sequence (CDS), 3′ UTR and stop codon, with small subsets located in the 5′ UTR and start codon (Fig. 1B, C). Notably, the density of m^6^A peaks in the 3′ UTR increased over time, and the representative motif sequence of m^6^A sites also underwent slight modification (Fig. 1B–D). Next, conjoint analysis of m^6^A RIP and RNA-seq was used to identify the differentially m^6^A-methylated and expressed molecules and some representative genes were marked (Fig. 1E). These m^6^A-tagged genes were found to be mainly enriched in biological processes related to signal transduction, transcriptional regulation and cell differentiation (Fig. 1F). The signaling pathways as transforming growth factor-β (TGF-β) and Rap1 were identified in KEGG pathway analysis (Additional file 1: Fig. S1A).

The establishment of m^6^A marks relies on the riboswitch from the methyl donor SAM to SAH conducted by the methyltransferase complex. We next evaluated the methylation potential and expression pattern of m^6^A-related molecules during DPSC mineralization. The concentrations of the methyl metabolite (SAH) were increased after induction, while the methyl donor (SAM) and methylated potential (SAM/SAH ratio) showed no significant variation (Fig. 1G). RNA-seq analysis showed that the relative gene expression of *METTL3* was markedly increased on days 7 and 14 of induction among all the m^6^A-related molecules (Additional file 1: Fig. S1B). Regarding methyltransferases, the mRNA and protein levels of METTL3 also increased after odontogenic induction and peaked on day 14, while METTL14 exerted low protein expression levels, and WTAP showed no obvious trend (Fig. 1H, I). Taken together, these data suggest that METTL3 is the key player in m^6^A modification during DPSC mineralization.

### METTL3 is required for functional DPSC differentiation

**Fig. 2:**
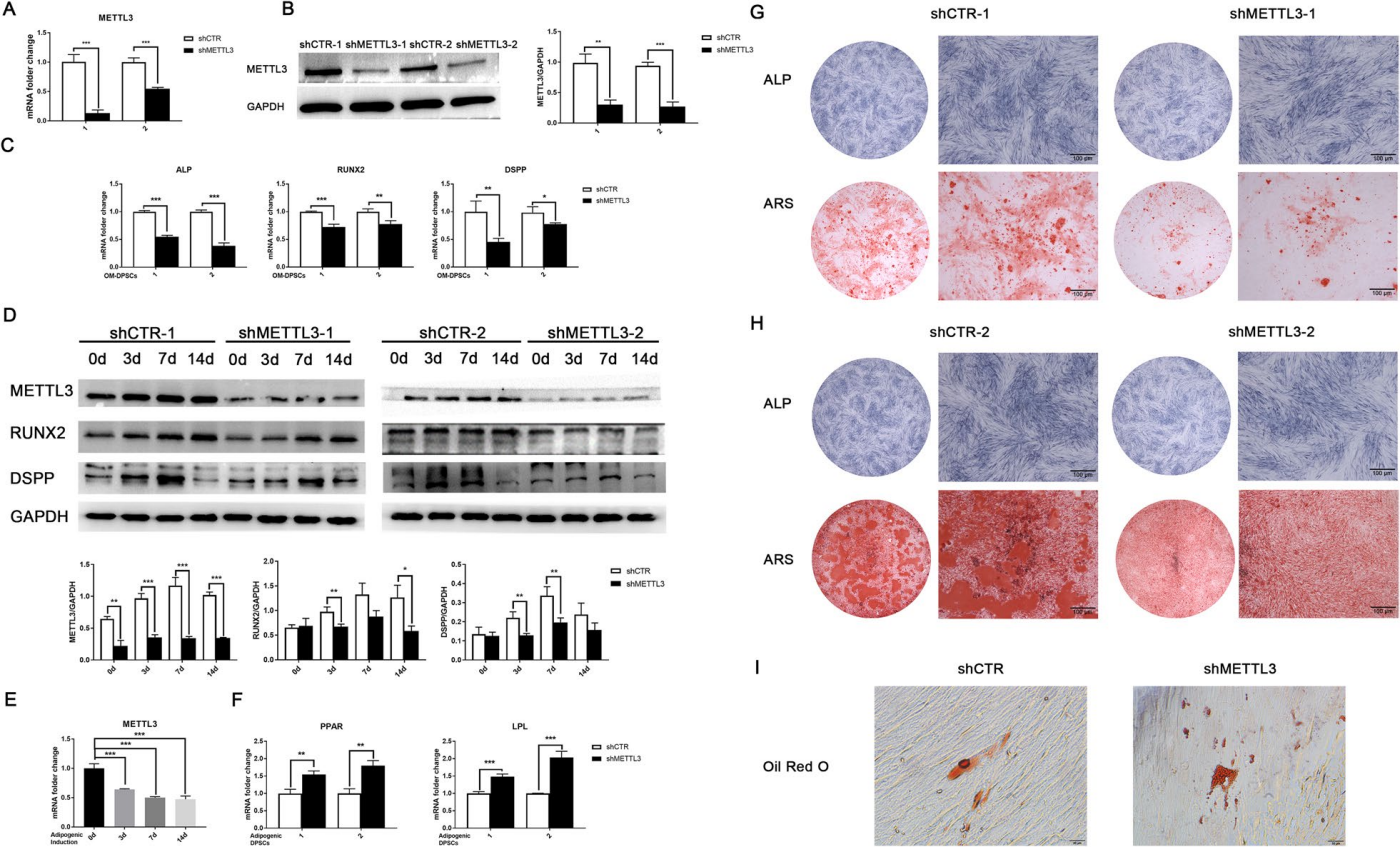
METTL3 is required for functional DPSC differentiation. **A** Two independent shRNA lentiviruses were used to inhibit *METTL3* expression, as confirmed by qPCR. **B** Western blotting detected the protein expression of METTL3 in DPSCs after METTL3 shRNA treatment (n = 3). **C** qPCR analysis of the mRNA expression of *ALP*, *RUNX2* and *DSPP* in differentiated DPSCs (n = 3). **D** The protein expression levels of METTL3, RUNX2 and DSPP in METTL3 knockdown DPSCs under odontogenic induction were evaluated by western blotting. **E** The mRNA expression level of *METTL3* in DPSCs after adipogenic induction for 0, 3, 7, and 14 days. **F** The mRNA expression of *PPAR* and *LPL* after adipogenic induction as detected by qPCR (n = 3). **G** The odontogenic differentiation of shMETTL3-1 and shCTR-1 lentivirus-transduced DPSCs was evaluated by ALP and ARS staining (n = 5). **H** The differentiation capacity of shMETTL3-2- and shCTR-2-transduced DPSCs after induction. **I** Representative images of Oil Red O staining in METTL3 knockdown DPSCs. OM-DPSCs: DPSCs induced by odontogenic medium. Significance was determined via ANOVA or Student’s t test; the data are presented as the mean ± SD (n ≥ 3). *p < 0.05. **p < 0.01. ***p < 0.001

To identify the potential impact of METTL3 deletion on DPSC differentiation, two shRNA lentiviral vectors were used to knockdown METTL3 expression and both sufficiently suppressed the mRNA and protein expression by more than half (Fig. 2A, B). *ALP, RUNX2* and *DSPP* were also downregulated at the mRNA level (Fig. 2C). The protein expression of RUNX2 was significantly reduced after 3 and 14 days of induction, and DSPP was also suppressed on days 3 and 7 (Fig. 2D). METTL3 knockdown led to weaker ALP activity and less mineralized nodule formation after 7 and 14 days of odontogenic induction (Fig. 2G, H). These results indicated that METTL3 inhibition impaired the odontogenic differentiation of DPSCs. Meanwhile, the expression of *METTL3* was suppressed during adipogenic induction (Fig. 2E). METTL3 inhibition enhanced the mRNA expression of *Lipoprotein Lipase (LPL)* and *Peroxisome Proliferator Activated Receptor (PPAR)* and promoted lipid droplet formation (Fig. 2F, I). Overall, METTL3 inhibition compromised odontogenesis but promoted adipogenesis, implying a critical role of m^6^A methylation and METTL3 in controlling DPSC differentiation.

### METTL3 overexpression enhances the mineralization of DPSCs

**Fig. 3:**
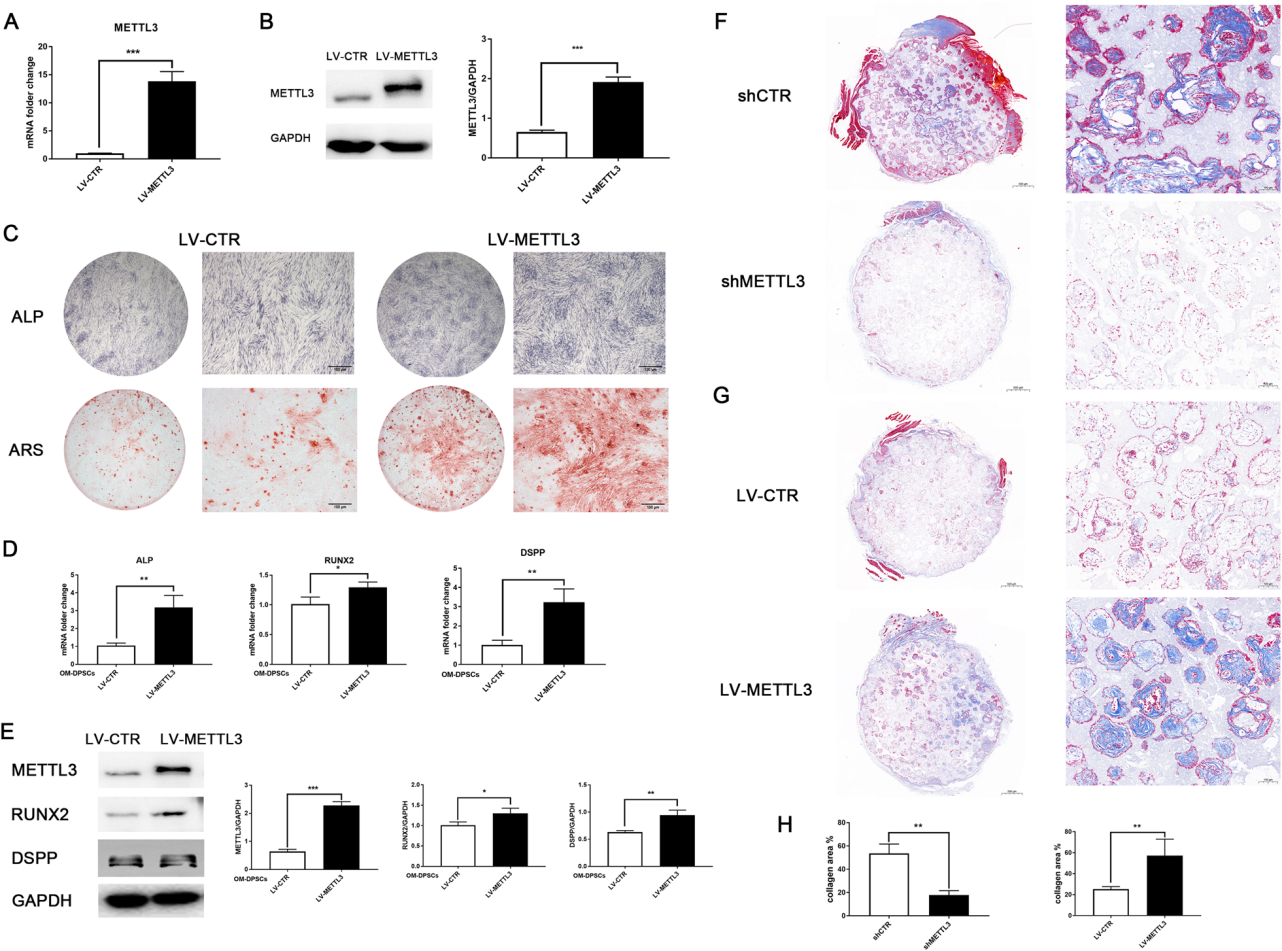
METTL3 overexpression enhances the mineralization of DPSCs. **A** Lentivirus was constructed to overexpress METTL3, as confirmed by qPCR. **B** Western blotting detected the protein expression of METTL3 in DPSCs after METTL3 overexpression. **C** The odontogenic differentiation of DPSCs after METTL3 overexpression was evaluated by ALP staining and ARS staining (n = 5). **D** qPCR analysis of the mRNA expression levels of *ALP*, *RUNX2* and *DSPP* in METTL3-overexpressing OM-DPSCs. **E** The protein expression levels of METTL3, RUNX2 and DSPP were assayed by western blotting (n = 3). **F** Masson trichrome staining of the composites of β-TCP/HA scaffolds and DPSCs treated with METTL3 shRNA after subcutaneous transplantation in nude mice for 4 weeks (n = 5). **G** Subcutaneous transplantation of DPSCs transduced with METTL3 overexpression and control lentivirus (n = 5). **H** Collagen fibers and newly formed mineralized tissue were stained blue and evaluated by ImageJ. Significance was determined via two-tailed Student’s t test; the data are presented as the mean ± SD (n ≥ 3). *p < 0.05. **p < 0.01. ***p < 0.001

Considering the negative effect of METTL3 inhibition, we hypothesized that METTL3 overexpression would benefit DPSC mineralization. The elevated mRNA and protein levels of METTL3 upon overexpression lentivirus transfection were confirmed (Fig. 3A, B). METTL3 overexpression induced ALP activity and calcium nodule formation (Fig. 3C). The favorable effect of METTL3 overexpression was also evidenced by the increased mRNA expression of *ALP*, *RUNX2*, and *DSPP* and protein expression of RUNX2 and DSPP (Fig. 3D, E). These results support the promoting effect of METTL3 overexpression on DPSC odontogenic differentiation and matrix mineralization.

To evaluate the role of METTL3 in DPSC mineralization in vivo, we conducted subcutaneous transplantation of β-TCP/HA scaffolds with DPSCs in BALBc nude mice. DPSCs were subjected to METTL3 knockdown or overexpression prior to the incubation with β-TCP/HA scaffolds. Hematoxylin and eosin staining was performed to evaluate cellularity alteration (Additional file 1: Fig. S2) and Masson-trichrome staining assess extracellular matrix production. Immature mineralized tissue and collagen fiber (blue staining) formation were reduced by approximately two-thirds in the METTL3 knockdown DPSC groups compared to the control groups (Fig. 3F, H). Moreover, METTL3 overexpression enhanced collagen tissue formation by more than twofold after 4 weeks (Fig. 3G, H), indicating the therapeutic potential of METTL3 in DPSC differentiation and mineralized tissue formation.

### Dynamic m^6^A modification of *NOG* orchestrates the differentiation stage

**Fig. 4:**
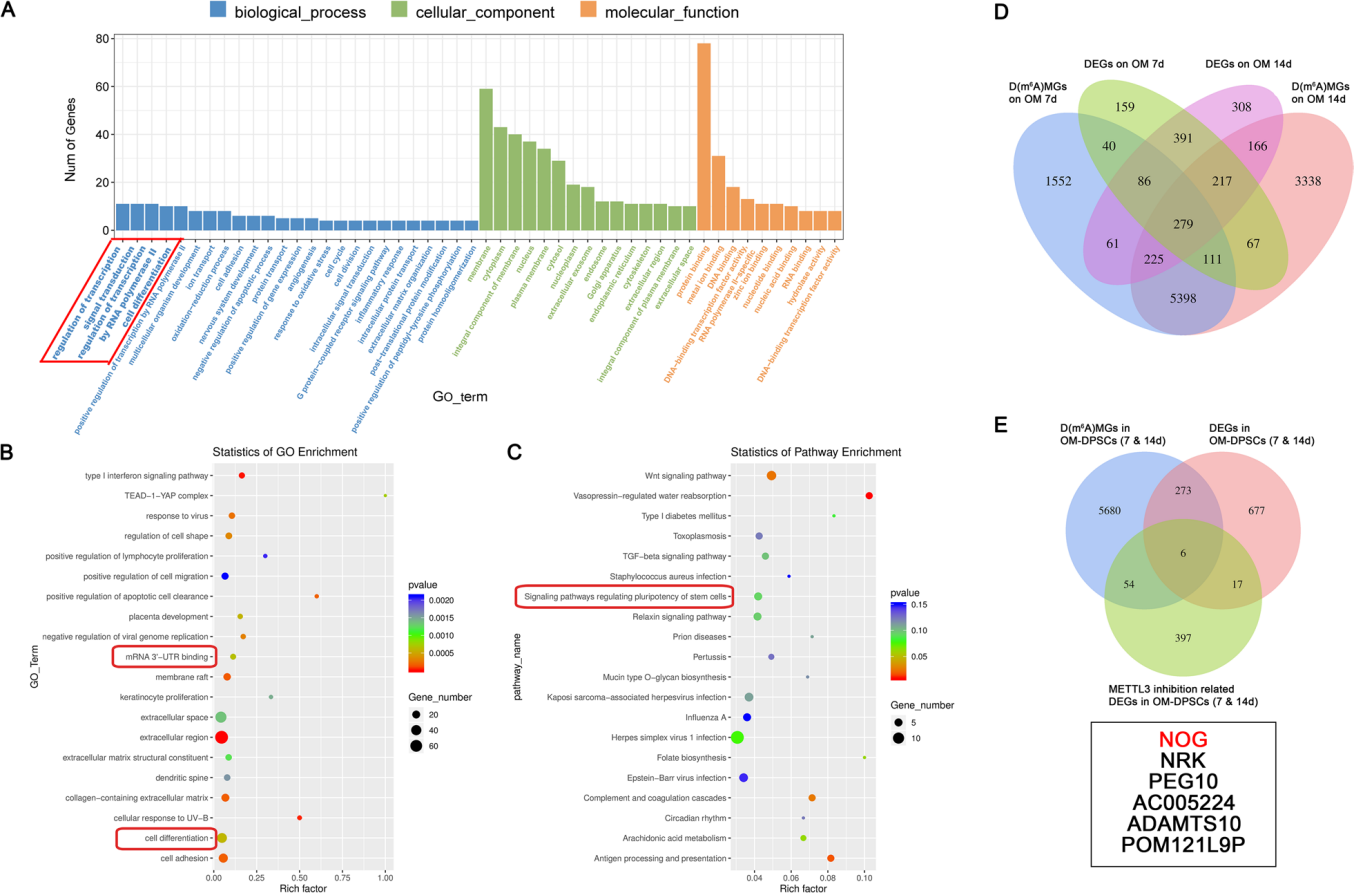
*NOG* is a target gene of METTL3-mediated m^6^A modification. **A** The top terms identified by Gene Ontology enrichment analysis of overlapping DEGs in METTL3-knockdown OM-DPSCs after induction for both 7 and 14 days. **B** Scatter plots of the top items in Gene Ontology enrichment. **C** The enrichment analysis of KEGG pathways. **D** The differentially m^6^A methylated and expressed genes in the m^6^A-mRNA profile after induction for 7 and 14 days by m^6^A RIP & RNA sequencing. **E** Venn diagram showing overlapping genes between DEGs in transcriptome sequencing after METTL3 knockdown and m^6^A-methylated transcripts during DPSC mineralization. DEGs: differentially expressed genes; D(m^6^A)MGs: differentially m^6^A methylated genes

To dissect the potential targets of METTL3-mediated m^6^A modification in DPSC differentiation, transcriptome sequencing was used to screen the gene expression profile after METTL3 deletion in OM-DPSCs (Additional file 1: Fig. S3). GO analysis showed that these DEGs were mainly related to mRNA 3′ UTR binding, signal transduction and cell differentiation (Fig. 4A, B), which corresponds to the elevated m^6^A accumulation in the 3′ UTR during DPSC mineralization. Signaling pathways regulating the pluripotency of stem cells, TGF-β and Wnt signaling pathways were also enriched in the KEGG pathway analysis (Fig. 4C). In the m^6^A-RIP and transcriptome sequencing of DPSC mineralization, 279 genes with significant differences in both m^6^A peaks and expression were identified (Figs. 1E, 4D). Next, we filtered the potential m^6^A target genes of METTL3 by taking the intersection of these 279 genes and the shMETTL3-related DEGs in both the early and late stages of differentiation (Fig. 4E). Among the six candidate targets, *NOG* is a well-known inhibitor of bone morphogenetic protein (BMP) signaling and the downstream Smad pathway.

**Fig. 5:**
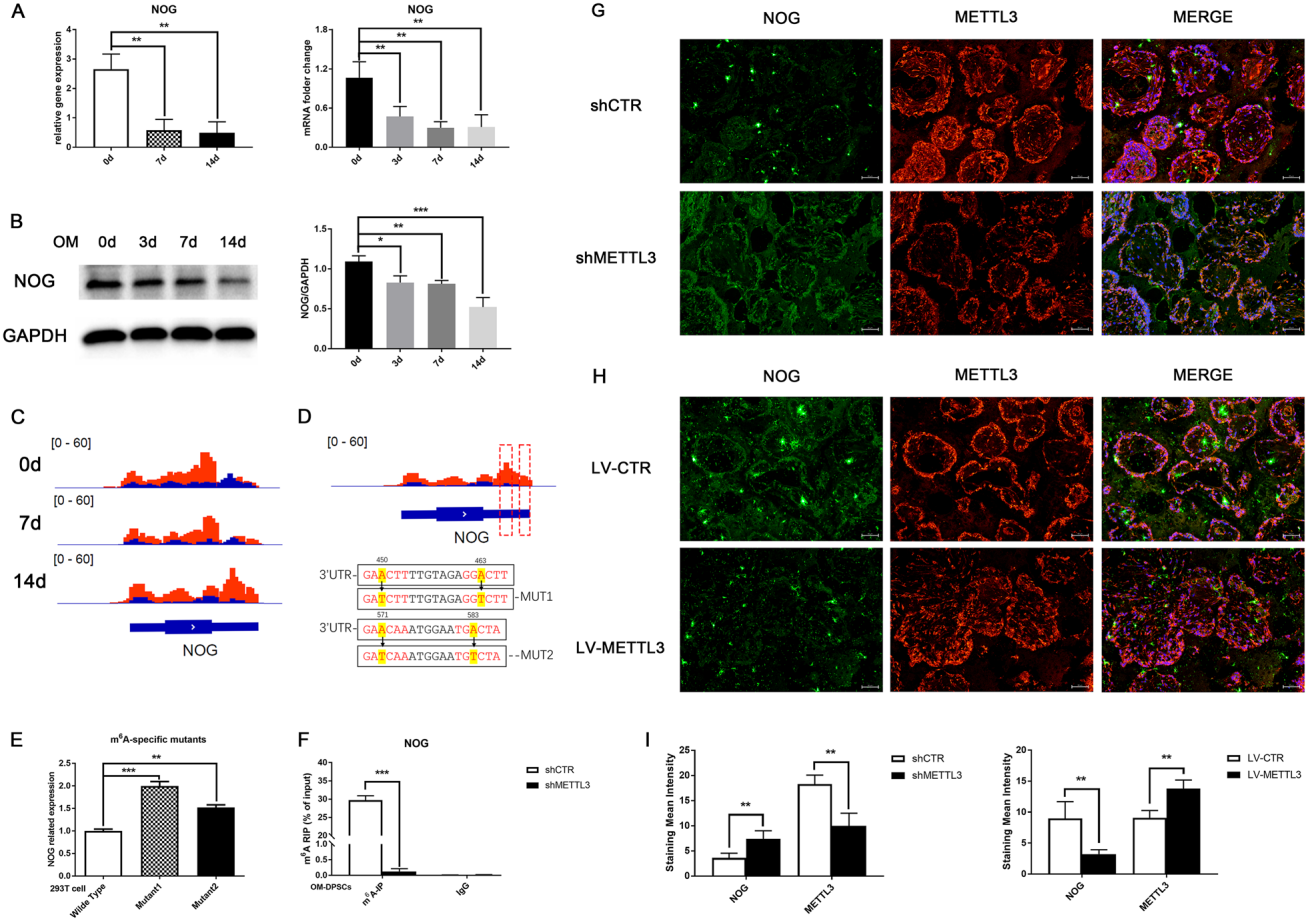
Dynamic m^6^A modification of *NOG* orchestrates the differentiation stage. **A** The relative gene expression and mRNA expression of *NOG* during DPSC differentiation. **B** The protein expression level of NOG detected by western blotting. **C** Visual data of high-confidence m^6^A peaks enriched in *NOG* mRNA detected by m^6^A RIP-seq. **D** Details of vectors containing fragments of the *NOG* 3′ UTR with the wild-type m^6^A motif or two independent m^6^A mutants (A-to-T mutation). The numbers (450, 463 and 571, 583) represent the positions of the m^6^A sites relative to the 3′ UTR. **E** qPCR assay of the mRNA expression of *NOG* in 293T cells transduced with the wild-type or m^6^A-mutant vector (n = 3). **F** m^6^A RIP-qPCR demonstrated the m^6^A deposition alteration in *NOG* mRNA after METTL3 inhibition. **G** Immunofluorescence staining showed the METTL3 and NOG expression in the composites of β-TCP/HA scaffolds with METTL3-knockdown DPSCs after subcutaneous transplantation in nude mice for 4 weeks. **H** Subcutaneous transplantation of DPSCs transduced with METTL3 overexpression and control lentivirus. **I** The expression level of METTL3 and NOG evaluated by imageJ. Significance was determined via ANOVA or Student’s t test; the data are presented as the mean ± SD (n ≥ 3). *p < 0.05. **p < 0.01. ***p < 0.001

The gene and protein expression levels of NOG were significantly reduced by half in DPSCs upon odontogenic induction, which was consistent with the transcriptome sequencing data (Fig. 5A, B). Immunofluorescence staining showed that the cytoplasmic expression of NOG was significantly suppressed after induction (Additional file 1: Fig. S4A). Moreover, the colocalization coefficient of METTL3 and NOG was reduced in differentiated DPSCs (Additional file 1: Fig. S4B). Visualization of m^6^A RIP-seq data showed that high-confidence and specific m^6^A peaks in the 3′ UTR of *NOG* mRNA markedly increased after induction (Fig. 5C). The two ascending m^6^A peaks in the 3′ UTR of *NOG* appeared to be the center of four m^6^A sites after mapping the conserved motif sequence. To explore the potential impact of these m^6^A marks on gene expression, *NOG* expression vectors containing wild-type m^6^A sites (WT) and two A-to-T m^6^A mutations (MUT1 and MUT2) were constructed (Fig. 5D). Both m^6^A mutations resulted in increased mRNA expression of *NOG* compared with WT expression in 293 T cells (Fig. 5E). These data demonstrated that m^6^A tag accumulation in the 3′ UTR of *NOG* resulted in diminished gene expression, which is required for DPSC differentiation.

Next, we investigated the potential effect of METTL3 depletion on m^6^A-tagged *NOG*. The m^6^A methylation level of *NOG* decreased sharply after METTL3 knockdown in OM-DPSCs (Fig. 5F), indicating that the elevated m^6^A enrichment in *NOG* was mediated by METTL3. In the ectopic mineralization models, the composites of β-TCP/HA scaffolds with METTL3-knockdown DPSCs showed a remarkable upregulation in NOG expression (Fig. 5G, I), while the NOG secretion were significantly suppressed in METTL3-overexpression transfected composites after subcutaneous transplantation (Fig. 5H, I).

### Stage-specific NOG expression is regulated by METTL3

**Fig. 6:**
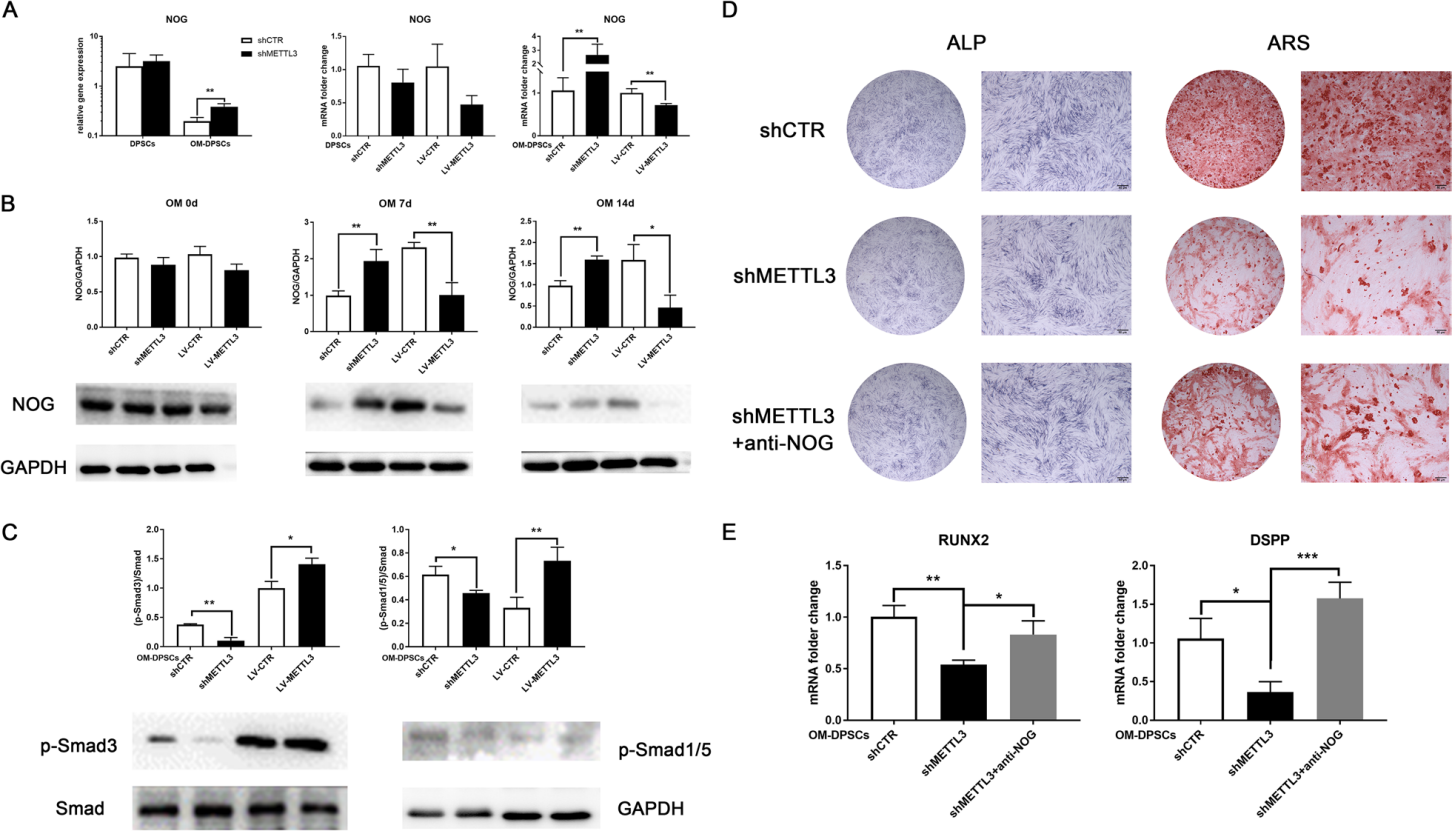
Stage-specific NOG expression is regulated by METTL3. **A** The relative gene expression and mRNA expression of *NOG* in differentiated and undifferentiated DPSCs. **B** The protein expression level of NOG after METTL3 alteration determined by western blotting (n = 3). **C** Activation of the downstream Smad signaling pathway was evaluated by measuring the protein expression of p-Smad3, p-Smad1/5 and Smad in OM-DPSCs. **D** ALP activity and calcium mineralization formation in METTL3-knockdown DPSCs treated with NOG blocking antibody (n = 3). **E** The *RUNX2* and *DSPP* mRNA expression levels assayed by qPCR. Significance was determined via ANOVA or Student’s t test; the data are presented as the mean ± SD (n ≥ 3). *p < 0.05. **p < 0.01. ***p < 0.001

NOG antagonizes BMP signaling and selectively inhibits BMP-related Smad pathway activity, which is essential for cell differentiation. Notably, METTL3 knockdown enhanced the relative expression of *NOG* in the differentiated state but not in the undifferentiated state, and qPCR confirmed the inhibitory effect of METTL3 on *NOG* mRNA expression in only OM-DPSCs (Fig. 6A). Additionally, METTL3 deletion significantly upregulated the protein expression of NOG in OM-DPSCs, and METTL3 overexpression exerted the opposite effect (Fig. 6B). Meanwhile, no stable trend was observed for NOG expression changes in undifferentiated DPSCs. These data suggested that METTL3 regulated m^6^A-tagged NOG expression to orchestrate the differentiation process. Furthermore, we evaluated the downstream signal transduction of NOG by assessing phosphorylation of Smad1/3/5 [24]. METTL3 deletion suppressed the phosphorylation of Smad3 and Smad1/5, while METTL3 overexpression enhanced the activation of the Smad pathway in differentiated DPSCs (Fig. 6C). Consistently, the phosphorylation level of Smad3 were dramatically inhibited in METTL3-knockdown DPSCs with β-TCP/HA scaffolds, while significantly enhanced in METTL3-overexpression composites in the ectopic mineralization models (Additional file 1: Fig. S5).

The impaired ALP activity and calcium mineralization formation induced by METTL3 inhibition were significantly upregulated after neutralizing NOG protein with a blocking antibody (Fig. 6D), as was the mRNA expression of *RUNX2* and *DSPP* (Fig. 6E). These data demonstrated that neutralizing excess NOG secretion can partially rescue insufficient odontogenic differentiation of METTL3-deleted DPSCs.

### METTL3 programs NOG destabilization via poly(A) tail shortening

**Fig. 7:**
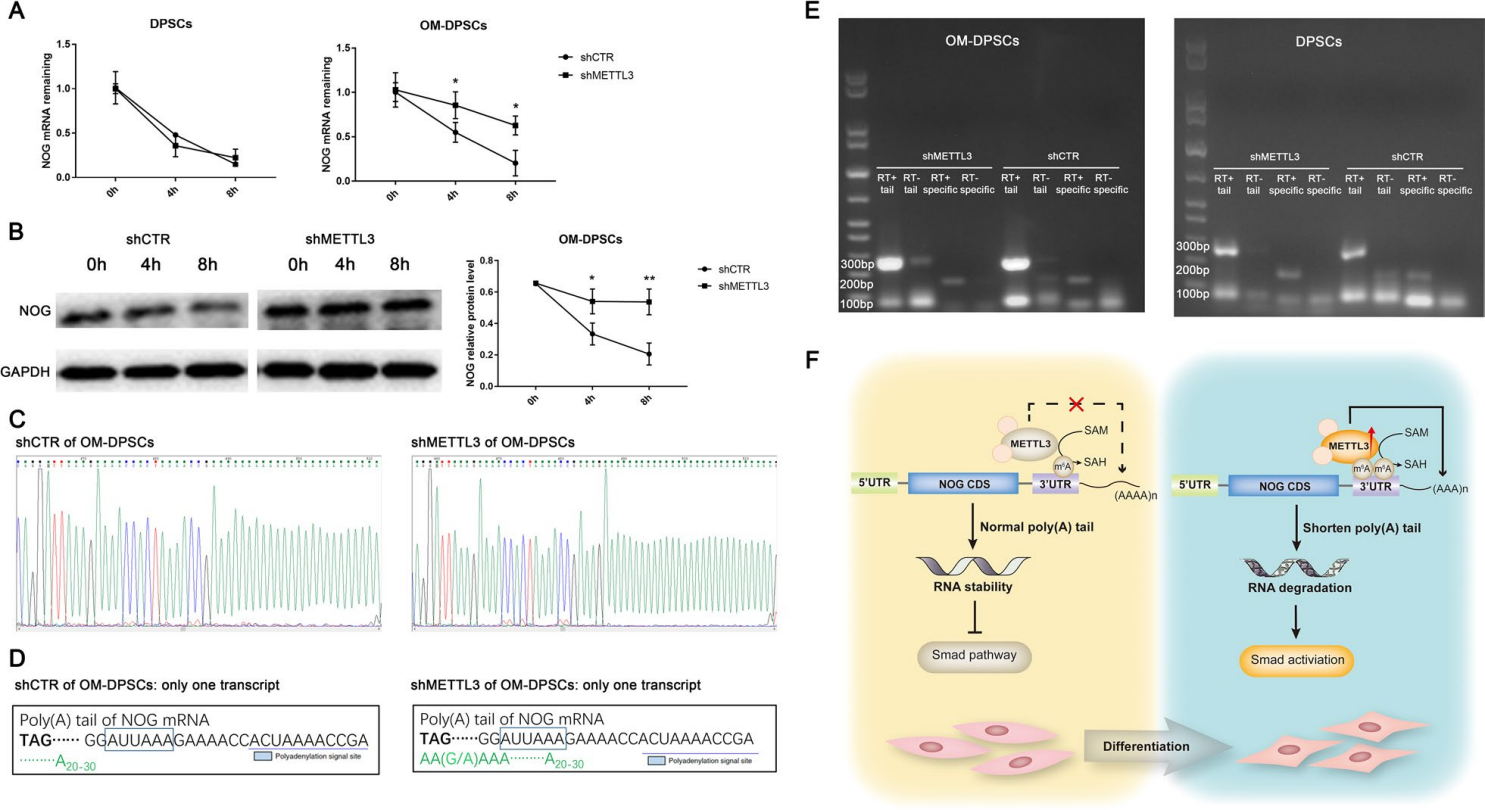
METTL3 programs NOG destabilization via poly(A) tail shortening. **A** The remaining *NOG* mRNA in differentiated and undifferentiated DPSCs treated with actinomycin D was quantified by qPCR. **B** Protein translation was blocked by cycloheximide, and the remaining expression level of NOG was detected by western blotting (n = 3). **C** 3′-RACE characterization of the polyadenylation site and 3′ UTR information of *NOG*. **D** Diagram of 3′-RACE analysis of *NOG* mRNA. **E** The poly(A) tail length of the *NOG* transcript in METTL3 knockdown DPSCs was assayed by poly(A) tail length measurement. **F** Schematic of the m^6^A modification machinery in DPSC mineralization. Significance was determined via ANOVA or Student’s t test; the data are represented as the mean ± SD (n ≥ 3). *p < 0.05. **p < 0.01. ***p < 0.001

Clustered m^6^A hallmarks in the 3′ UTR around the stop codon mainly influence the stability and localization of RNA. RNA stability assays with actinomycin D to suppress transcription showed that METTL3 knockdown increased the half-life of *NOG* mRNA in differentiated DPSCs (Fig. 7A). Furthermore, in the undifferentiated state, the mRNA decay of *NOG* was not significantly decelerated (Fig. 7A). Then, we blocked protein translation and synthesis by cycloheximide. The protein degradation of NOG in differentiated DPSCs was also suppressed after METTL3 inhibition (Fig. 7B). Consistent with previous data, METTL3 specifically restricted the stabilization and promoted the degradation of m^6^A-tagged NOG in differentiated DPSCs.

The poly(A) tail determines the mRNA stability and translation initiation of most eukaryotic mRNAs. Transcriptome sequencing suggested the presence of only one transcript of *NOG* without alternative splicing, which is in line with the transcript reported in the National Center for Biotechnology Information (NCBI) database (reference sequence: NC_000017.11). 3′-RACE analysis was conducted to obtain the 3′ UTR sequence information of *NOG* after METTL3 depletion. The 3′-RACE results showed no obvious alterations in the polyadenylation site or 3′ UTR length, while the number of A residues appeared to be increased in OM-DPSCs (Fig. 7C, D). Traditional RACE might result in PCR bias in the poly(A) tail due to the use of universal adaptor primers and nested amplification [22]. The poly (A) tail length was further quantified by PCR with *NOG*-specific primers after random oligomer addition of guanosine and inosine residues. The poly(A) tail was dozen bps longer after METTL3 knockdown in differentiated DPSCs, but this difference was not detected in undifferentiated DPSCs (Fig. 7E). These data support that METTL3 exerts temporal control over NOG stabilization to orchestrate DPSC differentiation (Fig. 7F).

## Discussion

Emerging evidence has proven that m^6^A RNA methylation is a critical epitranscriptomic mechanism that permits additional specificity and plasticity to the transcriptome [25]. Here, we revealed the dynamic and unique m^6^A mRNA landscape in DPSC mineralization, and elevated m^6^A marks in the 3′ UTR of certain transcripts are required for transcriptional prepatterning. METTL3 was identified as the essential m^6^A modulator in regulating DPSC differentiation. Furthermore, increasing m^6^A hallmarks in the 3′ UTR restricted the gene expression of NOG during DPSC mineralization. METTL3 mediated the m^6^A modification of NOG and promoted its degradation via poly(A) tail shortening in a stage-specific manner. The present study addressed a critical role of m^6^A modification in the temporal control of DPSC differentiation and provided new insight into the transcriptional coordination of stem cell regulation.

RNA m^6^A deposition is redundant in the consensus motif RRm^6^ACH ([G/A/U][G > A]m^6^AC[U > A > C]), which is enriched in the CDS and 3′ UTR of RNA transcripts [8, 26]. During embryonic cortical neurogenesis, m^6^A-methylated transcripts are enriched in biological processes, such as neural stem cells, the cell cycle, and differentiation, which are essential to control the transcriptome composition of different stages [25]. We characterized the dynamic and unique m^6^A landscape in DPSC mineralization, and the m^6^A-mRNA profile was mainly related to transcriptional regulation and cell differentiation. Moreover, the increasing total m^6^A content and m^6^A distribution in the 3′ UTR might result from a pronounced elevation of METTL3 expression. METTL3 was reported to participate in tooth root development by modulating translational efficiency [27]. The inhibition of DPSC proliferation and osteogenesis by METTL3 knockdown was associated with an impaired glycolytic pathway [28]. METTL3 is also involved in bone mesenchymal stem cell (BMSC) differentiation and function [16, 29]. METTL3 depletion in BMSCs impaired osteogenic differentiation, while METTL3 overexpression partly abrogated the induction of osteoporosis in mice [16]. Consistent with the current literature, METTL3 inhibition comprised DPSC differentiation, and METTL3 overexpression facilitated DPSC mineralization, indicating therapeutic potential. There are several in vivo models available to verify the regulatory mechanism of DPSC differentiation, such as dentin–pulp complex regeneration in situ and ectopic transplantation of DPSCs [30, 31]. Ectopic mineralization models were used in this study and subcutaneous transplantation in immunocompromised mice supported that METTL3 is a positive regulator of DPSC differentiation and mineralized tissue formation. More evidences from orthotopic models are needed to support the therapeutic application in vital pulp procedures and DPSC-based therapy.

Various transcripts and signals are tagged in a timely manner by m^6^A modification, which in turn controls proper development and differentiation. METTL3-mediated m^6^A modification regulates the expression of some osteogenic markers and other related genes involved in bone metabolism [32]. Parathyroid hormone (PTH)/Pth1r, TGF-β/SMAD, WNT and other signaling pathways are modulated by m^6^A marks, which are essential in the cellular differentiation and cancer development [16, 33, 34]. Here, NOG and downstream Smad pathway were identified as the target of METTL3-mediated m^6^A modification during DPSC differentiation. NOG is a key player in ectoderm development, and its disruption can lead to organogenesis defects such as craniofacial defects and hypoplastic teeth [35, 36]. Noggin is capable of binding and inactivating members of the TGF-β superfamily proteins as BMPs, subsequently blocking BMP-induced Smad pathway activation [37]. BMSC osteogenesis and DPSC odontogenesis are regulated by NOG via the downstream Smad1/5 signaling pathway [38, 39]. We found that the m^6^A peaks in the 3′ UTR of *NOG* mRNA increased during DPSC mineralization, which restricted its gene expression. METTL3 inhibited m^6^A-tagged NOG expression and promoted its degradation in differentiated DPSCs. Consistent with our data, m^6^A modification modulates RNA degradation and gene expression in neural stem cells, which is a critical epigenetic mechanism in the temporal control of neurogenesis [25]. m^6^A signaling clustered in the 3′ UTR is mainly responsible for cytoplasmic events related to RNA stability and translation [40–42], and METTL3 can independently read and modulate m^6^A marks in the 3′ UTR of certain transcripts [40]. Taken together, these findings suggest that m^6^A modification dynamically modulates the stability of specific transcripts, which is required for the transcriptional prepatterning of DPSC mineralization.

Poly(A) tails are 150–250 adenosine nucleotides acquired by the end of the 3′ UTR in the nucleus that subsequently undergo deadenylation in the cytoplasm. The length of a poly(A) tail changes throughout the lifetime of mRNA and has essential effects on its stability, degradation and translation [43]. In the global transcriptome, transcripts with a longer poly(A) tail possess a longer average mRNA half-life [23]. The deadenylation of shorter poly(A) tails can cause RNA decay or translational defects [44]. A recent study also noted the correlation between m^6^A marks and poly(A) tail regulation. The transcriptional dynamics of certain genes are related to differences in poly(A) tail length via m^6^A modification and deadenylase complexes [45]. m^6^A signaling is capable of controlling RNA structural switching and RNA‒protein interactions [46]. METTL3 and WATP can modulate RNA stabilization in an m^6^A-HuR-dependent manner [47, 48]. The m^6^A reader YTH N^6^-methyladenosine RNA binding protein (YTHDF) 2 is reported to directly interact with the CCR4-NOT complex. YTHDF3 can recruit the poly(A) specific ribonuclease subunit (PAN) 2-PAN3 complex, contributing to its deadenylation and degradation [49, 50]. Nonadenosine residues, such as G modifications, are also related to high quality and delayed degradation of the poly(A) tail [51, 52]. In our study, the temporal control of NOG stabilization by METTL3 relied on poly(A) tail shortening in the differentiation stage. Further studies are needed to identify the specific mechanism of how m^6^A marks lead to shortened poly(A) tails in DPSC differentiation.

The osteo/odontogenic differentiation of DPSCs and tertiary dentin formation are of particular interest in relation to dental repair. Identifying the key signaling in DPSC differentiation and mineralized matrix formation, and recapitulating these processes in clinical strategies could preserve pulp vitality. In the present study, we demonstrated that dynamic m^6^A RNA methylation is essential for heightened transcriptional coordination during DPSC differentiation. METTL3-mediated m^6^A marks tag the 3′ UTR of *NOG* and inhibit its stabilization via poly(A) tail regulation in a stage-specific manner. The present study identifies a critical role of METTL3-mediated m^6^A methylation in the temporal control of cell fate transition and sheds light on the epitranscriptomic machinery of m^6^A-dependent poly(A) tail regulation in transcriptional dynamics.

## Supplementary Information

**Additional file 1: Additional Table 1：** The sequence of primers used in qPCR. **Additional Figure 1:** KEGG enrichment analysis of m^6^A-tagged transcripts and the relative expression of m^6^A-related genes during DPSC mineralization. **Additional Figure 2:** H&E staining of the composites of β-TCP/HA scaffolds and DPSCs. **Additional Figure 3:** Volcano plots of differentially expressed genes in OM-DPSCs after METTL3 inhibition. **Additional Figure 4:** Immunofluorescence staining of METTL3 and NOG in DPSCs. **Additional Figure 5:** Immunofluorescence staining of p-Smad3 and Smad in the composites of β-TCP/HA scaffolds with METTL3-interfering DPSCs.

## Data Availability

The datasets used and analyzed during the current study are available from the corresponding author on reasonable request.
