# Supplementary material for: Stage-specific requirement for METTL3-dependent m6A modification during dental pulp stem cell differentiation
Source: J Transl Med. 2022 Dec 16;20:605. doi: 10.1186/s12967-022-03814-9 (PMC9756505; doi:10.1186/s12967-022-03814-9)
Supplement: Supplementary file 1 — Additional file 1: Additional Table 1： The sequence of primers used in qPCR. Additional Figure 1: KEGG enrichment analysis of m6A-tagged transcripts and the relative expression of m6A-related genes during DPSC mineralization. Additional Figure 2: H&E staining of the composites of β-TCP/HA scaffolds and DPSCs. Additional Figure 3: Volcano plots of differentially expressed genes in OM-DPSCs after METTL3 inhibition. Additional Figure 4: Immunofluorescence staining of METTL3 and NOG in DPSCs. Additional Figure 5: Immunofluorescence staining of p-Smad3 and Smad in the composites of β-TCP/HA scaffolds with METTL3-interfering DPSCs. [file 12967_2022_3814_MOESM1_ESM.pdf]

Additional Materials

Stage-specific requirement for METTL3-dependent m^{6}A modification during dental pulp stem cell differentiation

Additional Table

Additional Table 1: The sequence of primers used in qPCR

glyceraldehyde-3-phosphate dehydrogenase	*GAPDH*	F	TCAACAGCGACACCCACTC
		R	GCTGTAGCCAAATTCGTTGTC
methyltransferase 3	*METTL3*	F	GAGGAGTGCATGAAAGCCAG
		R	GGCCTCAGAATCCATGCAAG
methyltransferase 14	*METTL14*	F	GACGGGGACTTCATTCATGC
		R	CCAGCCTGGTCGAATTGTAC
WT1 associated protein	*WTAP*	F	ACGCAGGGAGAACATTCTTG
		R	CACACTCGGCTGCTGAACT
alkaline phosphatase	*ALP*	F	CCAAAGGCTTCTTCTTGCTG
		R	CCACCAAATGTGAAGACGTG
RUNX family transcription factor 2	*RUNX2*	F	TCGCCAGGCTTCATAGCAAA
		R	GGCCTTGGGTAAGGCAGATT
dentin sialophosphoprotein	*DSPP*	F	ATATTGAGGGCTGGAATGGGGA
		R	TTTGTGGCTCCAGCATTGTCA
peroxisome proliferator activated receptor	*PPAR*	F	TGGAGCCCAAGTTTGAGTTT
		R	CAGGGCTTGTAGCAGGTTGT
lipoprotein lipase	*LPL*	F	CAAGAGTGAGTGAACAAC
		R	AATTATGCTGAAGGACAAC
noggin	*NOG*	F	CGAGATCAAAGGGCTAGAGTTC
		R	GACCACAGCCACATCTGTAA
noggin (m^{6}A RIP-qCR)	*NOG*	F	CGAGCGAGATCAAAGGGCTA
		R	CGACCACAGCCACATCTGTA
noggin-FLAG	*NOG-FLAG*	F	GACTACAAGGACGACGACG
		R	GGCGGATGTGGAGATAGTG

Additional Figures:

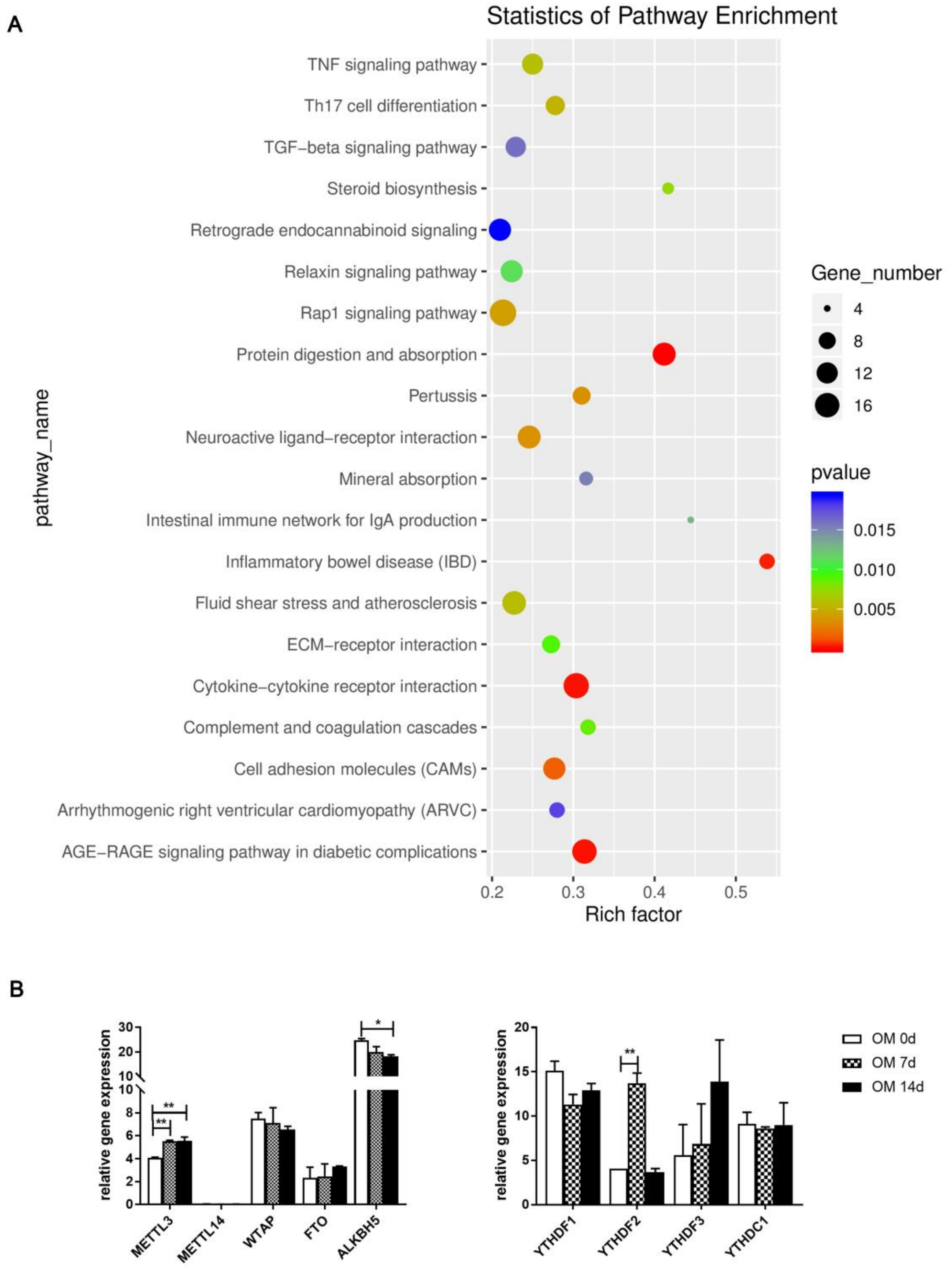

***Additional Figure 1*:** A, KEGG enrichment analysis of differentially m^{6}A methylated and expressed transcripts in DPSCs after odontogenic differentiation. B, The relative expression of m^{6}A-related genes during DPSC mineralization in transcriptomic sequencing. Significance was determined using one-way analysis of variance (ANOVA) and post-hoc Dunnett tests: *$p < 0.05$, **$p < 0.01$, and ***$p < 0.001$.

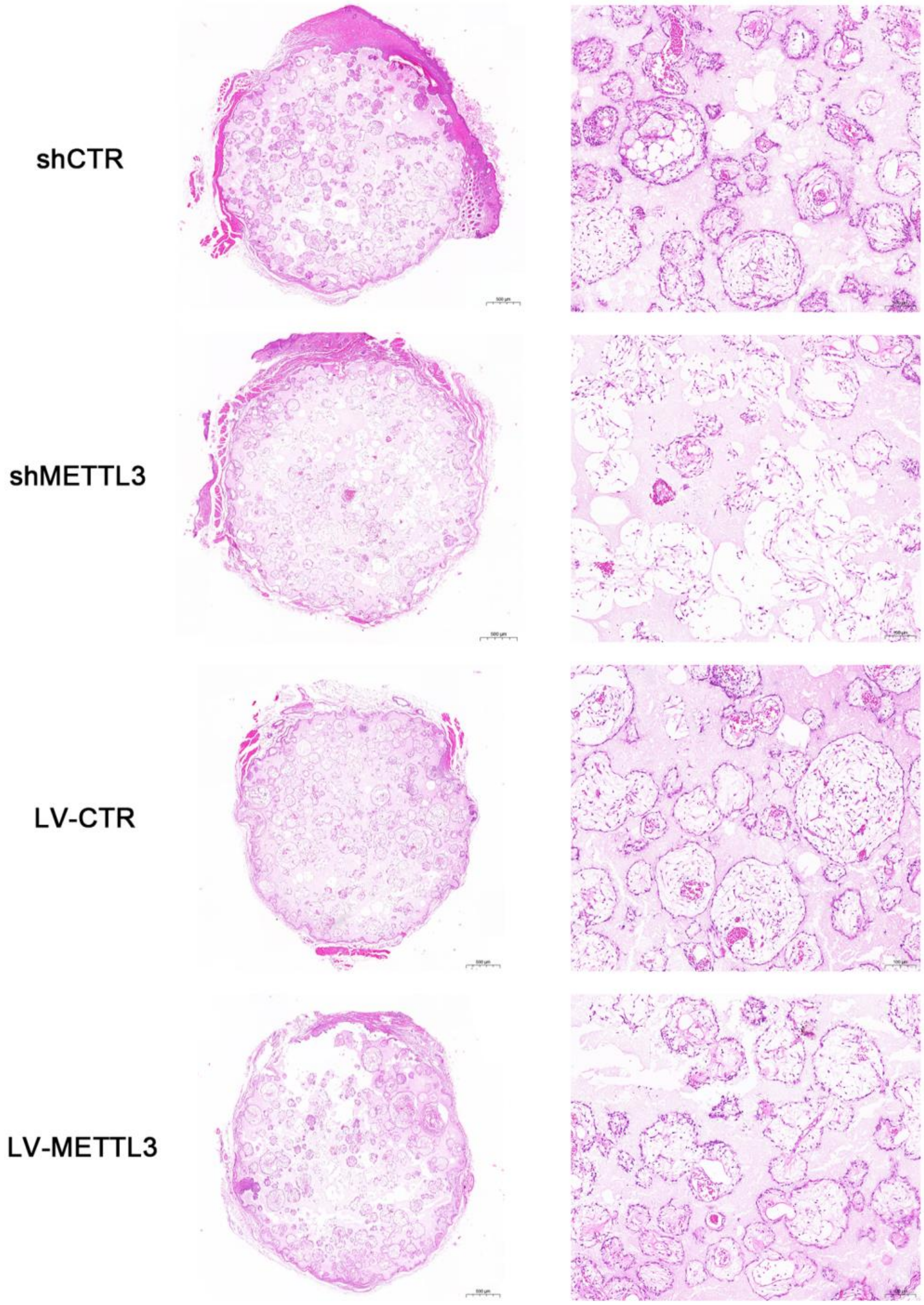

Additional Figure 2**:** Hematoxylin and eosin staining of the composites of β-TCP/HA scaffolds and DPSCs transfected with METTL3 knockdown or overexpression lentiviral vectors after subcutaneous transplantation of nude mice for 4 weeks.

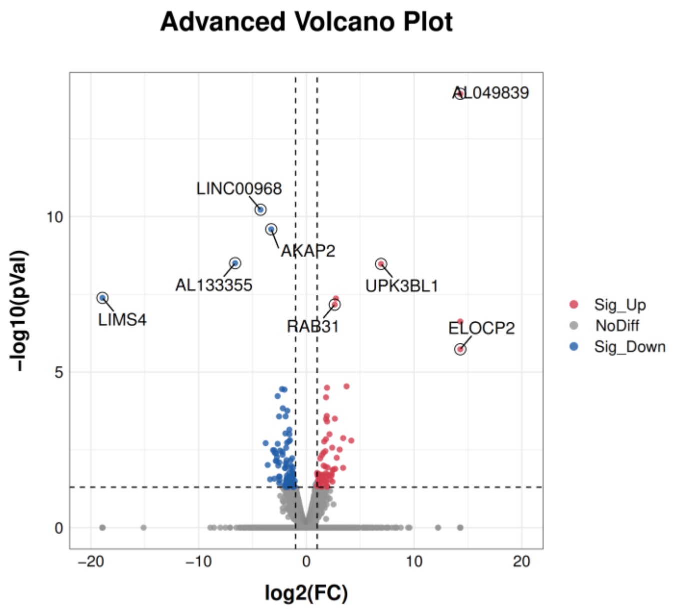

Additional Figure 3: Volcano plots of DEGs in OM-DPSCs after METTL3 inhibition by transcriptome sequencing (vertical lines represent >2.0-fold-change, horizontal lines for *p* values <0.05). Red dots indicate upregulated genes, and blue dots indicate downregulated genes.

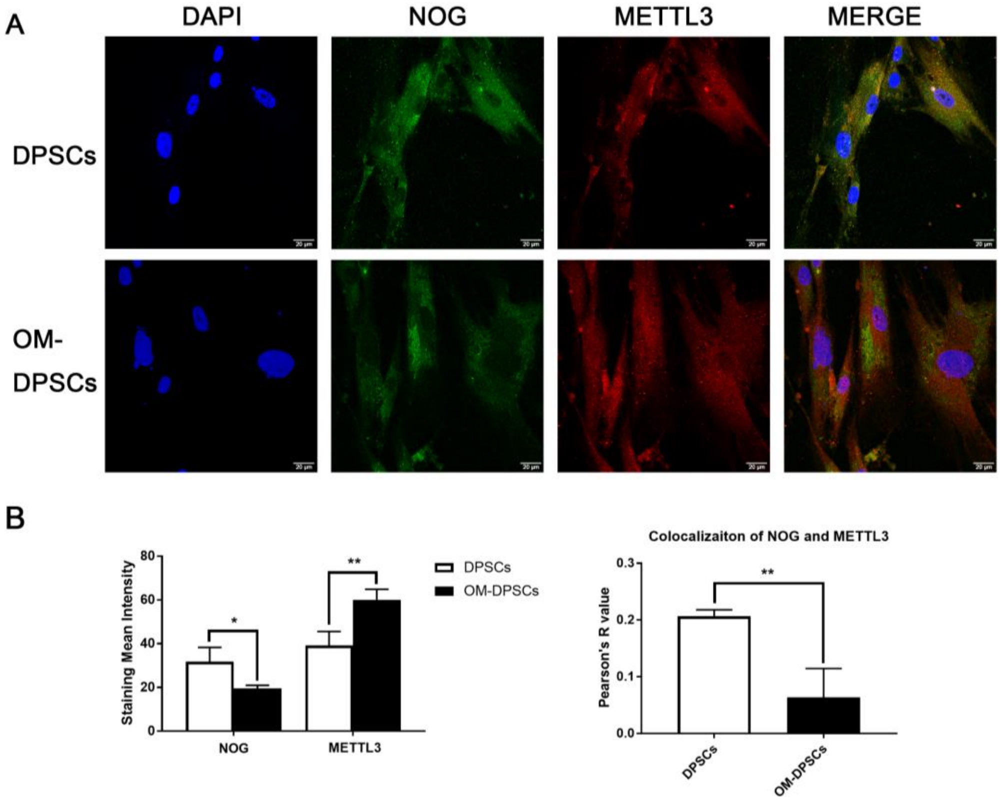

Additional Figure 4: A, Immunofluorescence staining showed the expression and location of METTL3 (red fluorescence) and NOG (green fluorescence) in undifferentiated and differentiated DPSCs. B, The expression level and colocalization coefficient of METTL3 and NOG evaluated by imageJ. Significance was determined via ANOVA or Student's t test; the data are presented as the mean ± SD (n≥3). **p* < 0.05. ***p* < 0.01. ****p* < 0.001.

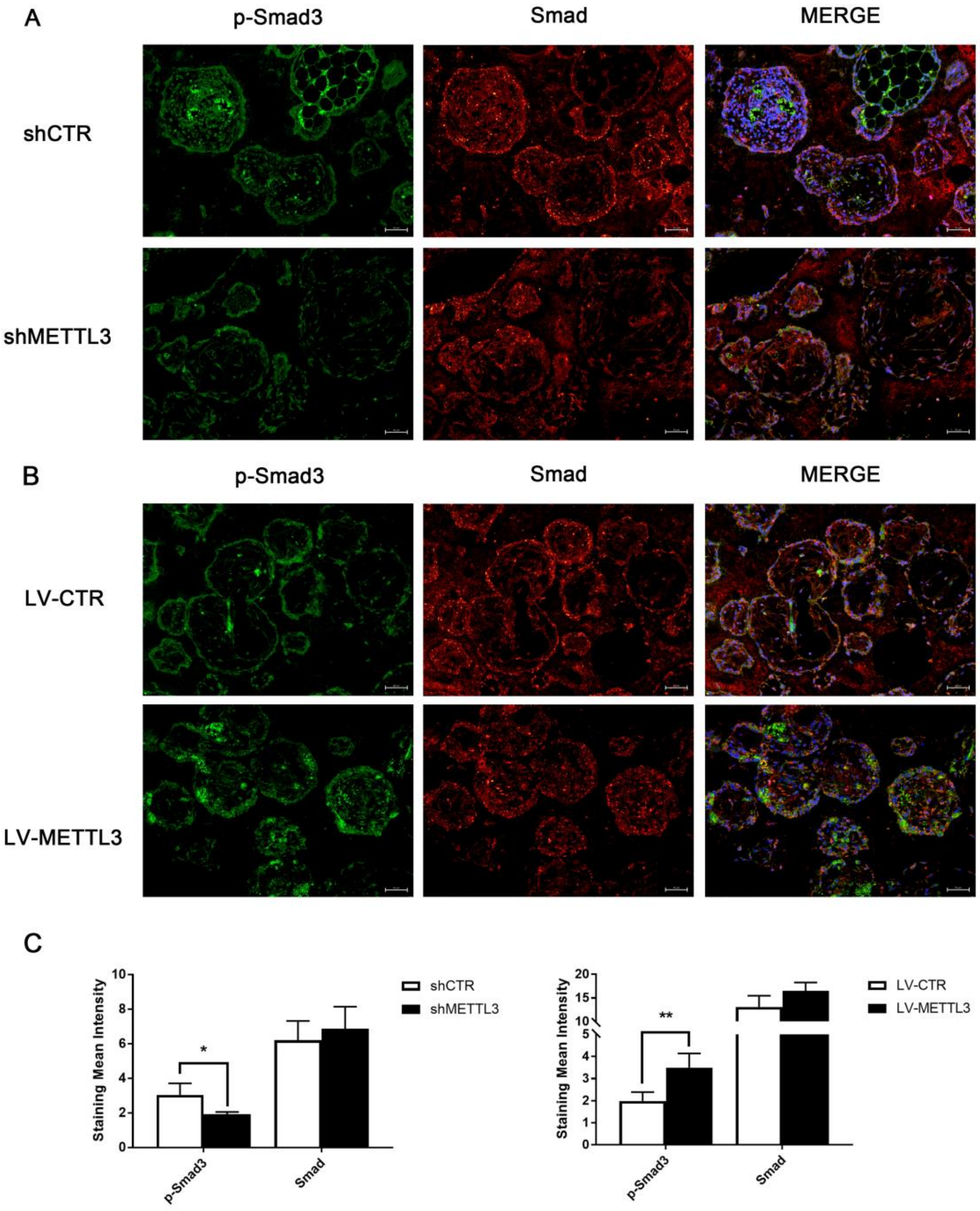

Additional Figure 5: A, Immunofluorescence staining showed the p-Smad3 and Smad expression in the composites of β-TCP/HA scaffolds with METTL3-knockdown DPSCs after subcutaneous transplantation in nude mice for 4 weeks. B, Subcutaneous transplantation of DPSCs transduced with METTL3 overexpression and control lentivirus. C, The expression level of p-Smad3 and Smad evaluated by imageJ. Significance was determined via ANOVA or Student's t test; the data are presented as the mean ± SD (n≥3). *p < 0.05. **p < 0.01. ***p < 0.001.
